# The role of spinal neurons targeted by corticospinal neurons in central poststroke neuropathic pain

**DOI:** 10.1111/cns.14813

**Published:** 2024-06-17

**Authors:** Fenqqi Fan, Tianze Yin, Biwu Wu, Jiajun Zheng, Jiaojiao Deng, Gang Wu, Shukun Hu

**Affiliations:** ^1^ Department of Pain, Yueyang Hospital of Integrated Traditional Chinese and Western Medicine Shanghai University of Traditional Chinese Medicine Shanghai China; ^2^ Department of Neurosurgery and Neurocritical Care, Huashan Hospital Fudan University Shanghai China

**Keywords:** AAV1‐Cre, central poststroke pain (CPSP), corticospinal neurons, mechanical allodynia, post‐synaptic targets, spinal interneurons

## Abstract

**Background:**

Central poststroke pain (CPSP) is one of the primary sequelae following stroke, yet its underlying mechanisms are poorly understood.

**Methods:**

By lesioning the lateral thalamic nuclei, we first established a CPSP model that exhibits mechanical and thermal hypersensitivity. Innocuous mechanical stimuli following the thalamic lesion evoked robust neural activation in somatosensory corticospinal neurons (CSNs), as well as in the deep dorsal horn, where low threshold mechanosensory afferents terminate. In this study, we used viral‐based mapping and intersectional functional manipulations to decipher the role of somatosensory CSNs and their spinal targets in the CPSP pathophysiology.

**Results:**

We first mapped the post‐synaptic spinal targets of lumbar innervating CSNs using an anterograde trans‐synaptic AAV1‐based strategy and showed these spinal interneurons were activated by innocuous tactile stimuli post‐thalamic lesion. Functionally, tetanus toxin‐based chronic inactivation of spinal neurons targeted by CSNs prevented the development of CPSP. Consistently, transient chemogenetic silencing of these neurons alleviated established mechanical pain hypersensitivity and innocuous tactile stimuli evoked aversion linked to the CPSP. In contrast, chemogenetic activation of these neurons was insufficient to induce robust mechanical allodynia typically observed in the CPSP.

**Conclusion:**

The CSNs and their spinal targets are required but insufficient for the establishment of CPSP hypersensitivity. Our study provided novel insights into the neural mechanisms underlying CPSP and potential therapeutic interventions to treat refractory central neuropathic pain conditions.

## Graphical abstract

Thalamic lesions lead to thalamic disinhibition and the overactivation of corticospinal neurons and their spinal targets, forming a feed‐forward loop that intensifies the central poststroke pain.

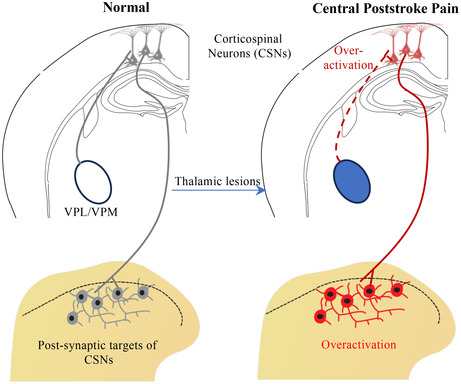

## 1 INTRODUCTION

The widespread occurrence and severe motor and somatosensory sequelae from strokes place them as a primary cause of disability in adults globally.^1^, ^2^ While rehabilitation training can promote locomotor recovery,^1^, ^3^, ^4^ a significant sensory after‐effect—namely, the central poststroke pain (CPSP)—remains challenging to manage. Thus, CPSP considerably hinders daily activities and diminishes the quality of life for stroke survivors.^5^

The earliest account of CPSP was documented by Dejerine and Roussy in 1906,^6^ who detailed the association between the thalamic stroke and a specific syndrome featured by severe pain and altered touch and deep sensory perception. Consistent with this, functional imaging in humans revealed that CPSP is commonly associated with strokes that affect the ascending spinothalamic tract and its thalamocortical projections.^5^, ^7^ Notably, a recent study discovered that CPSP patients with intact corticospinal tract (CST) exhibited enhanced cortical disinhibition, a condition that was linked with impaired life quality.^8^ This suggested that direct corticospinal control may exacerbate CPSP symptoms. The exact role and mechanism by which the CST and its downstream targets contribute to CPSP, however, remain to be elucidated.

The CST is a supraspinal descending pathway that directly connect the cortex with the spinal cord.^9^, ^10^ Damage to the CST axons leads to motor and sensory impairments after events such as stroke or brain and spinal cord injuries.^11^, ^12^, ^13^, ^14^ It has been documented that CST axons, which originate in various cortical areas, display distinct termination patterns within the spinal cord. For instance, those derived from the pre‐motor and motor cortices predominantly innervate the intermediate and ventral spinal cord, while those from the somatosensory cortices project to the spinal dorsal horn.^10^, ^15^, ^16^, ^17^ The heterogeneity of their cortical origins and spinal termination patterns suggest that spatially segregated corticospinal neuron subpopulations connect with distinct spinal neural circuits to orchestrate multiple sensorimotor behaviors.

Various spinal interneurons have been reported to receive direct input from the cortex, which are essential for coordinating diverse sensorimotor functions. For instance, Ch × 10‐expressing pre‐motor neurons receive descending input from the motor cortex and are involved in skilled reaching.^16^ Meanwhile, the dorsal horn excitatory interneurons expressing CCK are innervated by both low threshold mechanoreceptive afferents and somatosensory corticospinal descending axons.^18^, ^19^ These neurons are required for touch sensation and tactile neuropathic pain.^18^, ^19^ Despite of these insights, whether and how spinal interneurons, directly innervated by corticospinal neurons, are involved in the establishment and maintenance of CPSP hypersensitivity remains unexplored.

Here, we first generated a CPSP model that reliably produces prolonged mechanical allodynia. Mice with CPSP exhibited enhanced Fos activation following gentle touch in both corticospinal neurons and their post‐synaptic targets in the deep dorsal horn, as shown by using an AAV1‐Cre‐mediated anterogradely trans‐synaptic tracing strategy. Chronic inactivation of spinal interneurons targeted by CSNs blocked the development of CPSP. In addition, transient chemogenetic silencing of these neurons alleviated mechanical pain hypersensitivity and tactile stimuli induced aversion post‐lateral thalamic lesions. In contrast, animals with chemogenetic activation of these neurons exhibited no overt mechanical allodynia. Our study not only broadened our understanding of how the descending cortical pathway modulates spinal excitability, which is crucial for poststroke pain hypersensitivity, but also provided insights into the design of therapeutic targets to CPSP after traumatic brain injuries.

## 2 MATERIALS AND METHODS

### 2.1 Establishment of central poststroke pain model

All surgical procedures and behavioral measurements in this study were approved by the Use and Care of Animals Committee of Fudan University. To establish the CPSP model, C57 mice (P49‐64 with mixed sexes) were anesthetized using isoflurane (3% for induction and 1.5% for maintenance) and head‐fixed onto a stereotaxic frame (RWD). We then performed a stereotaxic injection of kainate or saline (0.3 μg in 1 × saline, 100 nL/site) into the lateral ventroposterior (VPL) thalamus nucleus (AP: −1.7, −2.0 mm; ML: 1.8 mm, depth: −3.5 mm). As a post hoc examination, mice used for pilot experiments were euthanized at 48 and 72 h post‐injection to examine lesions in the lateral thalamic nuclei with immunohistology of NeuN and Iba1. Consistent with previous reports,^20^, ^21^ we discovered comparable neuronal loss and inflammation at 48 or 72 h and then chose results from 48 h for histological analysis.

### 2.2 Measurement of mechanical and heat sensitivity

We used von Frey filaments to measure mechanical sensitivity of mice hindpaws. To start, mice were placed in a compact Plexiglas enclosure (dimensions: 7.5 cm by 7.5 cm by 15 cm) for a duration of 20 min or until they settled into a quiescent state. Subsequently, a sequence of von Frey filaments (Stoelting) was administered to the plantar aspect of the hindpaw. The response of the hindpaw withdrawal was noted for each filament, applied in ascending force order, conducted 10 times, and ensuring a minimum interval of 3 s for the mice to return to a state of rest between each test. The threshold for mechanical sensitivity, particularly for assessing punctate mechanical allodynia, was identified by the least forceful filament that consistently elicited a rapid withdrawal or escape behavior in response to five or more out of the 10 applications.

To measure heat sensitivity, mice were placed in a compact Plexiglas enclosure (dimensions: 7.5 cm by 7.5 cm by 15 cm) for a duration of 20 min or until they settled into a quiescent state. Infrared heat was then applied to hindpaw plantar surface with a Hargreaves apparatus (Ugo Basile). The hindpaw withdrawal latency was measured for individual mice.

### 2.3 Retrograde tracing of hindlimb corticospinal neurons

Protocols to retrograde tracing of hindlimb corticospinal neurons were adapted from our previous studies.^22^ In brief, C57 mice (P49‐P56 with mixed sexes) were anesthetized using isoflurane (3% for induction and 1.5% for maintenance) and positioned in a stereotaxic apparatus. After laminectomy, AAV‐Retro‐GFP (tilter adjusted to 1 × 10^13^ copies/mL, generated by BrainVTA, Wuhan, China) was injected into the lumbar dorsal horn spinal cord (L3‐L5) using the following coordinates: 0.2‐ and 0.5‐mm lateral to the midline; 0.4 mm beneath the dura, 100 nL per injection site.

### 2.4 Fos induction and immunohistochemistry with quantification

To induce Fos, at 21 days after intrathalamic lesion, mice with various treatment were habituated in the von Frey testing equipment. 0.16 g von Frey filament was applied to the contralateral hindpaw plantar at once every 10 s over 15 min.^23^ Mice received cardiac perfusion at 2‐h post‐repetitive von Frey filament stimuli.

To perform immunohistochemistry, brain or spinal tissues were post‐fixed in 4% paraformaldehyde (1×PBS), cryo‐protected with 30% sucrose overnight (1×PBS), and sectioned at 30 μm. The primary antibodies (4°, overnight) used in this study were a rabbit anti‐Fos [Cell Signaling (2250 s), 1:500]; a mouse anti‐NeuN [Millipore (MAB377), 1:200]; and a rabbit anti‐Iba1 [Thermo Fisher Scientific (10904‐1‐AP) 1:500]. The secondary antibody (room temperature, 2 h) used in this study was Alexa Fluor 555‐conjugated donkey anti‐rabbit (1:200). To estimate the neuronal loss caused by kainate injection, total NeuN^+^ cell number was divided by the area of the lateral thalamus nuclei (the VPL and VPM) and was quantified blindly using ImageJ2 (NIH). Raw data were then normalized to those from saline injection. To assessed microglial activation, we determined the ramification index of Iba1‐positive microglia by performing a morphometric analysis. In brief, we quantified the perimeter and area of individual Iba1‐positive microglial cell within the VPL/VPM area using ImageJ2 (NIH). The ramification index (R) was then calculated using the equation: *R* = (perimeter/area)/[2∙(π/area)^0.5], as described in.^24^ In both cases, data averaged from three sections crossing the rostral‐caudal VPL/VPM (AP: −1.2 to −2.2 mm) were calculated as a single data point for individual animal.

To determine the number of c‐Fos‐positive neurons in the cortex and lumbar spinal cord, five sections spanning the hindlimb S1, layer V in the hindlimb S1 as indicated with retrogradely labeled CSNs, or L3‐L5 spinal cord segments were blindly quantified for individual animal (ImageJ2, NIH). To estimate the activation of hindlimb CSNs, the proportion of Fos^+^/retrogradely labeled CSNs (GFP^+^) of total CSNs were quantified per section. To estimate Fos distribution in distinct laminae of L3‐L5 spinal cord segments, we identified borders of laminae structures according to the schema developed by Rexed et al.^25^

### 2.5 Mapping of hindlimb CSNs innervating spinal interneurons

To trans‐synaptic label spinal targets of distinct CSN subgroups, we injected AAV1‐Cre into different cortical regions of Ai14 (Jax:007914) mice with mixed sexes. In brief, adult Ai14 mice (6–8 weeks) with mixed sexes were anesthetized using isoflurane (3% for induction and 1.5% for maintenance) and head‐fixed onto a stereotaxic frame (RWD). AAV1‐Cre (tilter diluted to 1 × 10^13^ copies/mL, generated by BrainVTA, Wuhan, China) was then unilaterally injected into cortical areas where CSNs controlling forelimb and hindlimb are located. To label hindlimb CSNs, the coordinates used were AP: 0, −0.5, −1, −1.5 mm anterior to the bregma and ML: 2 mm lateral to the middle line, depth: 0.6 mm.^26^, ^27^ For individual site, 200 nL of viruses were injected.

To assess the distribution of post‐synaptic targets of hindlimb CSNs in the spinal cord, mice received cardiac perfusion at 3‐week post‐brain injection. The lumbar spinal cord was dissected out and sectioned at 30 μm using a cryostat machine. The number of Cre^+^ neurons in distinct laminae was blindly quantified/section. For individual animals, 10 sections crossing the L1‐L5 were used. Data were then averaged from multiple animals.

To assess Fos activation in spinal interneurons of hindlimb innervating CSNs, AAV1‐Cre was injected into the hindlimb cortical region in Ai14 animals. Three weeks later, mice received saline or kainate acid injection into the VPL. The Fos induction and examination were performed as described in the “Fos induction and immunohistochemistry with quantification” session. We calculated the ratio of Fos^+^tdTomato^+^ over total tdTomato^+^ neurons in the spinal cord/section in animals injected with saline or kainate acid following von Frey stimuli. For individual animals, five sections crossing the L3‐L5 were used. Data were then averaged from multiple animals.

### 2.6 Assessing Fos activation in spinal interneurons of hindlimb innervating CSNs

Ai14 animals whose hindlimb cortical region was injected with AAV1‐Cre or C57 mice with intersectional expression of mCherry or hM3Dq‐mCherry received cardiac perfusion (4% PFA in 1×PBS) at 60 min following tactile stimuli or CNO injection, respectively. The lumbar spinal cord was dissected out, post‐fixed in 4% PFA overnight, and sectioned at 10 μm using a cryostat machine (Leica). We then performed the in situ hybridization using the RNAscope® Multiplex Fluorescent Detection Kit v2 (Advanced Cell Diagnostics) following the manufacturer's guidelines. Probes used were *Cre* (cat# 312281), *Fos* (cat# 316921), and tdTomato (cat# 317041). We used a confocal laser‐scanning microscope (Zeiss 700) to obtain fluorescent images and then processed blindly. We calculated the ratio of *Fos* ^+^ *Cre* ^+^/*Cre* ^+^ or *Fos* ^+^ *tdTomato* ^+^ */tdTomato* ^+^ neurons in the spinal cord/section. For individual animals, five sections crossing the L1‐L5 were used. Data were then averaged from multiple animals.

### 2.7 Functional manipulation of spinal post‐synaptic targets of hindlimb CSNs

To functionally manipulate spinal targets of hindlimb CSNs, we performed intersectional stereotaxic injections. In C57 wildtype mice (6–8 weeks, mixed sexes), we first performed unilateral injection of AAV1‐Cre (tilter diluted to 1 × 10^13^ copies/mL, generated by BrainVTA, Wuhan, China) into the hindlimb sensorimotor cortex (coordinates and volumes see above). Two weeks later, we injected AAV2/8‐DIO‐mCherry (control, tilter diluted to 5 × 10^12^ copies/ml, BrainVTA, Wuhan, China) or AAV2/8‐DIO‐TeLC‐P2A‐EYFP (tilter diluted to 5 × 10^12^ copies/ml, generated by BrainVTA, Wuhan, China) or AAV2/8‐DIO‐hM3Dq‐mCherry (tilter diluted to 5 × 10^12^ copies/ml, generated by GenePharma, Shanghai, China) or AAV2/8‐DIO‐hM4Di‐mCherry (tilter diluted to 8 × 10^12^ copies/mL, generated by BrainVTA, Wuhan, China) into the contralateral dorsal horn spinal cord of the lumbar spinal cord L3‐L5 (for each segment, 150, 300, and 450 μm lateral to the midline, 250 μm beneath the dura, 100 nL/injection site).

Three weeks after the second injection, mice were subject for a battery of sensorimotor behavioral for baseline measurement. To chemogenetically activate or silence hindlimb CSNs or their targeted interneurons, CNO (1 mg/kg in saline) was intraperitoneally injected. Sensorimotor behaviors were assessed at 30 min post‐CNO administration. For hM3Dq‐mCherry/mCherry spinally injected group, animals received cardiac perfusion at 60 min post‐CNO administration to examine Fos activation in post‐synaptic targets of hindlimb CSNs by performing in situ hybridization.

### 2.8 Ground walking

Mice were placed in a narrow Plexiglas rectangle box to familiarize the device prior to the test day. At the test day, distinct joints were marked, and mice were video‐captured for their locomotion using a GoPro camera from the side view when they were walking the length of the box. Hindlimb weight support (height of lilac crest) and hindlimb stride length of mice with different treatment were then blindly measured.

### 2.9 Irregular ladder walking

The irregular ladder walking test was employed to evaluate skilled motor performance, which is dependent on cortical function.^11^, ^28^ The device consists of a one‐meter horizontal ladder with unevenly spaced rungs, elevated about 50 cm above the test table. Initially, mice were allowed to explore the apparatus to become accustomed to it. Subsequent to this familiarization, they underwent a training session on the second day to cross over the ladder until their performance achieved the plateau (a success rate about 85%–90%). Side view video recordings (Go Pro camera) of the sessions were made for post hoc blind analysis. The definition of a successful hindlimb step required the center of mouse's hindpaw to fully contact the rung. Any missed or slipped steps were recorded as unsuccessful. The success rate was quantified by the ratio of successful steps to the total number of steps taken.

### 2.10 Conditioned placement aversion assay

The conditioned placement aversion (CPA) assay was performed as previously described with modifications.^29^ In brief, the CPA setup featured two compartments, each measuring 10 × 10 × 15 cm—with one dark and the other white colored. A central rectangular gap (4 × 8 cm) was placed in between that allowed mice to freely explore both chambers. The apparatus was positioned over an elevated metal grid. Initially, on the first day, mice were allowed 15 min of free movement between the two chambers for a preliminary assessment. Typically, mice displayed a preference for the dark compartment. The conditioning spanned over 4 days, during when the rectangular gap was sealed. On each following mornings, mice were confined to the white chamber for 20 min. Conversely, on each following afternoons, mice were placed in the dark chamber where their contra‐lesional hindpaw received repetitive von Frey filament (0.16 g) stimulation for 20 min (5 s intervals). CNO (1 mg/kg in saline), if needed, was administrated at 30 min prior to every day's conditioning. On the sixth day, the passage was left open, and mice were once again allowed to move freely between both chambers for 15 min. A monitor system (Noldus) was placed on top of the apparatus to record mice locomotion trajectories. The aversion score was calculated as the change in time spent in the dark chamber between the initial and final tests, quantified in seconds (i.e., aversion score = time in dark chamber during pre‐test—time in dark chamber during post‐test).

### 2.11 Statistical analysis

In the conduction of all behavioral tests, animals of both sexes were randomized and distributed into different experimental groups. The evaluation of behavioral responses and the analysis of histological data were conducted in a blinded manner, as previously mentioned. Data for numerous graphs were displayed as mean ± standard error of the mean (SEM).

The assessment of data normality and variance uniformity was carried out using Stata software (version 12). For the statistical evaluations, we utilized a two‐tailed Student's *t*‐test for paired comparisons, one‐way ANOVA for single‐factor analysis, and two‐way ANOVA for repeated measures, with each followed by a Bonferroni post hoc test for multiple comparisons (using Prism 8.0 by GraphPad).

## 3 RESULTS

### 3.1 Establishment of a central poststroke pain model

**FIGURE 1:**
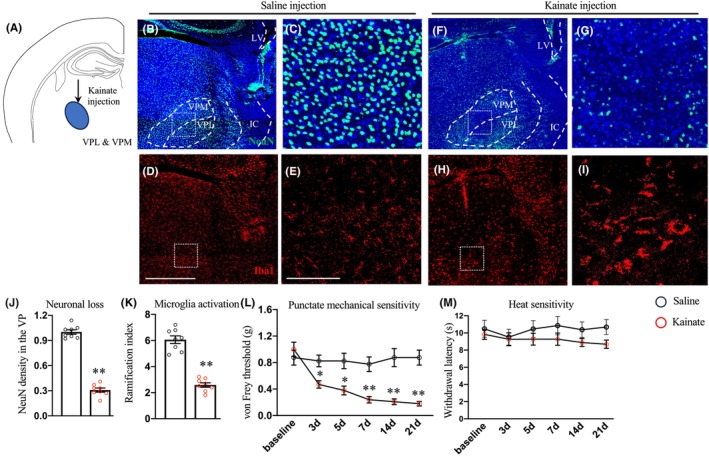
Sensory hypersensitivity post‐excitotoxic lateral thalamic lesion. (A) Cartoon schematic drawing of the experimental design. (B–I) Representative transverse images of the lateral thalamus stained with NeuN (green) (B, C, F, G) and Iba1 (red) (D, E, H, I) in mice injected with saline or kainate. Scale bars: 500 (D) and 100 (E) μm. Dashed square indicated areas with magnified images in C, E, G, and I. (J, K) Quantification of relative neuronal density (F) and ramification index (G) within the lateral thalamus in mice injected with saline (*n* = 8) or kainate (*n* = 8). (L, M) Measurement of mechanical (L) or heat (M) sensitivity at various time points following unilateral injection of saline (*n* = 8) or kainate (*n* = 8) into the lateral thalamus. **p < 0.01 and *p < 0.05, Student's *t*‐test (J, K) or one‐way ANOVA followed by the Bonferroni correction (L, M).

A large portion of patients with CPSP showed infractions in the lateral thalamic,^5^, ^30^ where somatosensory information is relayed from the spinal cord to the cortex. To simulate thalamic lesions observed in patients with CPSP, we unilaterally injected kainate into the thalamic ventral posterolateral nucleus (VPL)^21^, ^31^ (Figure 1A). After 48 h post‐injection, we observed significant neuronal loss, indicated by reduction of NeuN^+^ cells, within the VPL and the adjacent ventral posteromedial nucleus (VPM) (Figure 1B–G,J). Additionally, we discovered prominent inflammation within the lateral thalamus nuclei, evidenced by drastic changes in Iba1^+^ microglia ramification, confirming the induction of excitotoxic lesion (Figure 1D–K). Behaviorally, starting from the 3d following kainate injection, the punctate mechanical sensitivity was significantly reduced to a level around 0.16 g (Figure 1L), indicative of mechanical allodynia. Such mechanical pain hypersensitivity maintained for at least 3 weeks post‐injury (Figure 1L). In contrast, we did not observe heat hypersensitivity in this lesion model, consistent with previous observations (Figure 1M).^31^

### 3.2 Activation of somatosensory corticospinal neurons and spinal dorsal horn neurons by innocuous mechanical stimuli in animals with CPSP

The characteristic thalamocortical denervation and recent clinical observations made us to hypothesize that somatosensory cortical hypersensitivity contributed to pain hypersensitivity. To test this hypothesis, we applied repetitive punctate stimuli (0.16 g von Frey filament, 15 min) and examined Fos activity in various brain and spinal regions.

**FIGURE 2:**
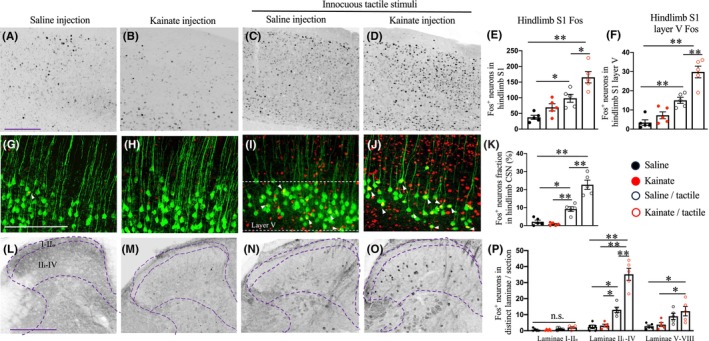
Fos activation by innocuous tactile stimuli post‐excitotoxic lateral thalamic lesion. Fos activity was assessed in the somatosensory cortex and spinal cord of saline (A, G, L, *n* = 5) or kainite‐injected animals (B, J, M, *n* = 5) or animals with saline (C, I, N, *n* = 5) or kainate (D, J, O, *n* = 5) injection following innocuous tactile stimuli. (A–D) Representative images of transverse sections of the hindlimb sensorimotor cortex stained for Fos in animals with different treatment. (E, F) Quantifications of Fos expression in total (E) or layer V (F) neurons in the hindlimb sensorimotor cortex in animals with different treatment. (G–K) Representative images of transverse sections of the hindlimb sensorimotor cortex stained for retrogradely traced corticospinal neurons (GFP) and Fos (G–J) with quantifications (K) of the percentage of Fos neurons in hindlimb corticospinal neurons. (L–P) Representative transverse images of spinal sections stained for Fos (L–O) with quantifications of Fos^+^ neurons in different spinal laminae (P). Scale bars: 200 μm. *, **, or no statistical differences, one‐way ANOVA followed by the Bonferroni correction.

We first assessed the somatosensory cortex, where tactile information from the spinal cord is conveyed and processed. Compared to naive mice, von Frey filament stimulation enhanced Fos activity in the hindlimb primary somatosensory cortex (hS1), particularly in layer V where corticospinal neurons reside (Figure 2A,C,E–K). The thalamic lesion alone had a minor effect on neuronal activation in the hS1or in layer V corticospinal neurons (Figure 2B,E,F,H,K). In contrast, in mice with lateral thalamus lesion, innocuous tactile stimuli significantly amplified Fos activity in the hS1, particularly in layer V (Figure 2D–F). Consistently, a substantial of Fos^+^ neurons were co‐localized with retrogradely labeled CSNs (Figure 2J,K), suggesting robust recruitment of CSNs upon innocuous tactile stimuli.

In the spinal cord, while saline injection alone, thalamic lesion alone, or von Frey filament stimulation alone induced sparse Fos activity, tactile stimuli in mice with lateral thalamic lesions evoked specific Fos activation in the deep dorsal horn (laminae II_I_‐IV), where CST axons terminate (Figure 2L–P). Taken together, these results suggested that lateral thalamic lesion led to overactivation of CSNs and characteristic patterns of spinal interneurons following innocuous mechanical stimulation.

### 3.3 Activation of CST innervated spinal interneurons by innocuous mechanical stimuli in animals with CPSP

The observed overlap in patterns of spinal neurons activated by CPSP and of the CST axon termination indicated that post‐synaptic targets of CSNs play a role in mechanical allodynia following intrathalamic damage. To test this, we first mapped the cortico‐spinal connectome by using an AAV1‐Cre‐based anterograde trans‐synaptic strategy.^32^, ^33^

**FIGURE 3:**
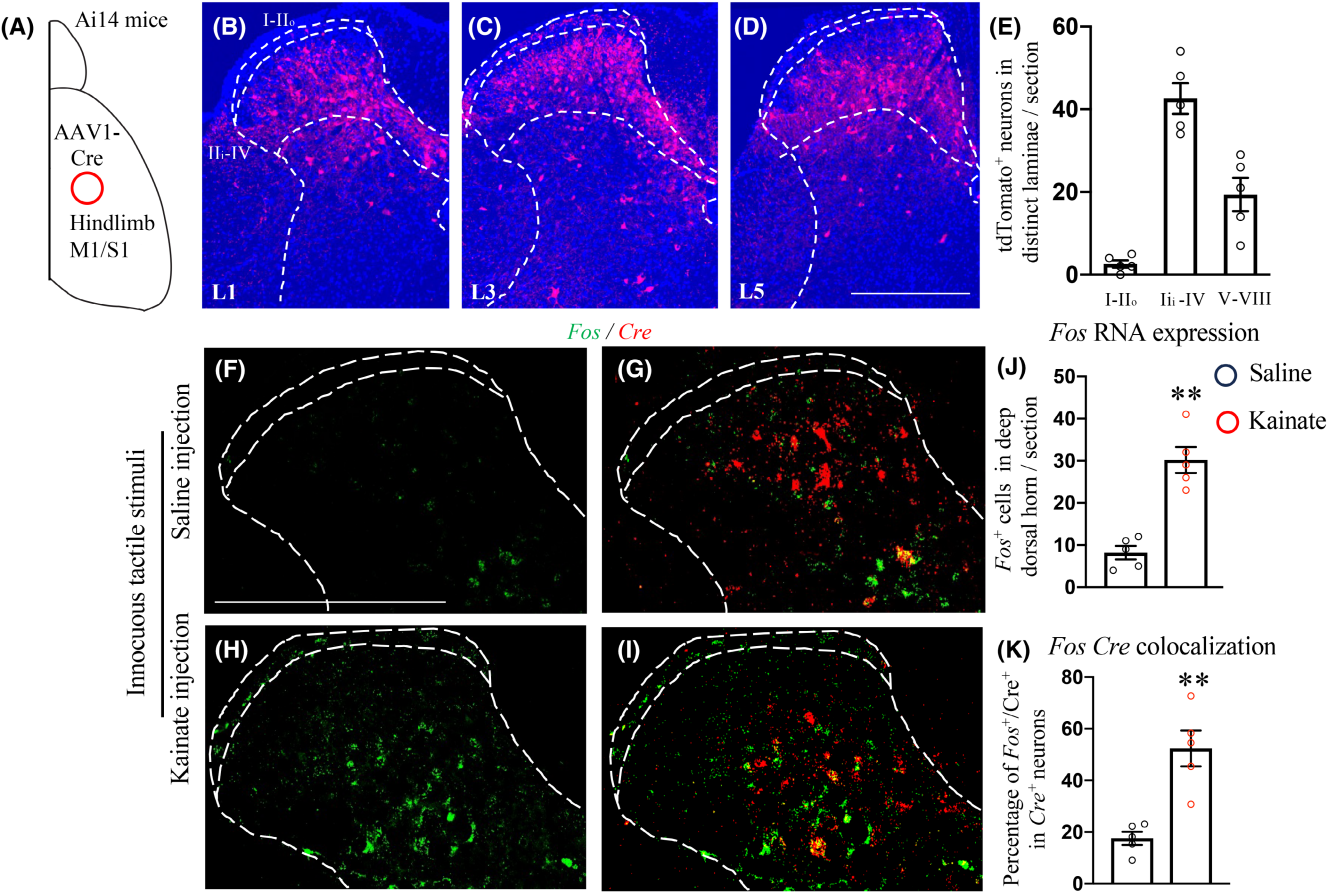
Activation of post‐synaptic targets of the CST in the lumbar spinal cord by innocuous tactile stimuli post‐excitotoxic lateral thalamic lesion. (A) Schematic diagram of the experimental design. (B–E) Representative images of transverse spinal sections at different lumbar segment showing anterogradely labeled tdTomato^+^ neurons (B–D) with quantifications of tdTomato neurons in different laminae (E, *n* = 5). (F–K) Representative images of fluorescent in situ hybridization of transverse spinal sections for *Fos* and *Cre* RNA expression from animals with saline (F, H, *n* = 5) or kainate (G, I, *n* = 5) injection following innocuous tactile stimuli with quantifications of Fos expression (J) and Fos proportion in CST targeted spinal interneurons (K). Scale bars: 500 μm. **p < 0.01, Student's *t*‐test.

In Ai14 mice, a Cre‐dependent tdTomato reporter strain, we stereotaxically injected AAV1‐Cre into the hindlimb sensorimotor cortex^26^, ^27^ (Figure 3A).

Three weeks post‐injection, we observed that spinal neurons innervated by hindlimb CSNs were highly enriched in the deep (laminae IIi‐IV) but not superficial (laminae I‐IIo) dorsal horn (Figure 3B–E), where the low‐threshold mechanical sensory afferents primarily terminate.^18^ In addition, less post‐synaptic targets of CSNs were identified in the intermediate and ventral spinal cord (laminae V‐VII) (Figure 3B–E), where pre‐motor neurons reside. The distribution pattern of hindlimb CSNs targeted spinal interneurons was consistent with their spinal projection patterns.^19^, ^34^

Consistent with Fos staining, repetitive von Frey stimulation following VPL lesion led to prominent increase of Fos expression in the deep dorsal horn (Figure 3F,H,J).

Importantly, a significant number of these activated dorsal horn interneurons were positive for anterogradely transported Cre, indicating they were direct recipients of cortical input (Figure 3G,I,K). These results suggested that the downstream targets of CSNs were activated in response to innocuous touch post‐VPL lesion.

### 3.4 Effects of chronic silencing of CST targeted spinal interneurons on CPSP hypersensitivity

**FIGURE 4:**
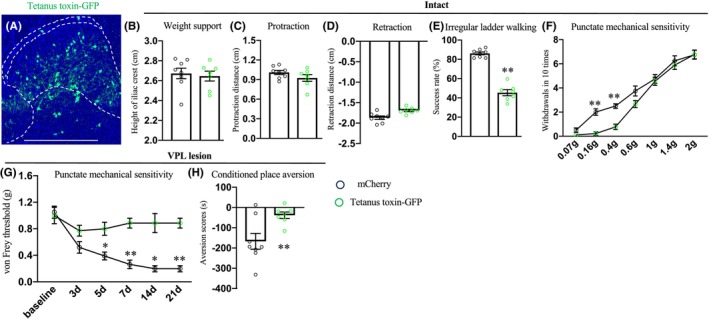
Effects of chronic inactivation of the CST targeted lumbar spinal interneurons on the development of CPSP. (A) A representative image of a transverse lumbar spinal dorsal horn stained with GFP, demonstrating intersectional expression of tetanus toxin (*n* = 7, experimental) in CST targeted lumbar spinal interneurons. Intersectional expression of mCherry (*n* = 8) was used as control. Scale bar: 500 μm. (B–F) Performance of ground walking (B–D), irregular ladder walking (E) task, and measurement of punctate mechanical sensitivity (F) in intact control and experimental mice. (G, H) Measurement of mechanical sensitivity at various time points (G) and aversion of tactile stimuli (H) in control and experimental mice receiving excitotoxic lateral thalamic lesions. (E and H) **p < 0.05, Student's *t*‐test. (F and G) **p < 0.01 or *p < 0.05, repeated measures of ANOVA followed by the Bonferroni correction.

To functionally dissect the role of spinal targets of the CST in CPSP hypersensitivity, we subsequentially injected (1) AAV2/1‐Cre into the sensorimotor cortex and (2) AAV2/8‐DIO‐ TeLC‐P2A‐EYFP into the contralateral spinal cord segments of C57 mice. This approach enabled the selective expression of the tetanus toxin light chain in spinal interneurons that receive direct inputs from the cortex (Figure 4A). Three weeks after spinal injection, mice exhibited no overt deficits in ground walking (Figure 4B–D). In contrast, their performance on an irregular ladder walking task, a measure of cortical‐dependent fine motor control^11^, ^28^, ^35^ was significantly compromised (Figure 4E). In addition, chronic inactivation of spinal neurons targeted by the CST reduced tactile responses to innocuous but not noxious mechanical stimuli (Figure 4F). Taken together, these results confirmed the effectiveness of the tetanus toxin in deactivating CST targeted spinal interneurons.

We then injected kainate into the lateral thalamus. In contrast to control mice, in which mechanical allodynia developed following intrathalamic injury, tetanus toxin‐based inactivation of post‐synaptic targets of the CST resulted in no mechanical hypersensitivity at multiple time points after intrathalamic lesion (Figure 4G). To determine whether silencing these spinal neurons also influenced the aversive aspect of pain, we conducted a touch‐evoked conditioned place avoidance assay. Unlike the control group, which displayed a strong aversion to their preferred chamber following innocuous stimuli, there was a significantly reduced aversion to von Frey filament stimulation when the post‐synaptic targets of corticospinal neurons were inhibited (Figure 4H). Thus, chronic inactivation of spinal neurons receiving cortical input was sufficient to prevent CPSP hypersensitivity.

### 3.5 Effects of transient chemogenetic silencing of post‐synaptic targets of the CST on CPSP hypersensitivity

Employing the same intersectional strategy, we next introduced excitatory or inhibitory DREADDs into spinal interneurons receiving inputs from CSNs, enabling us to temporarily modulate their activity.^36^

**FIGURE 5:**
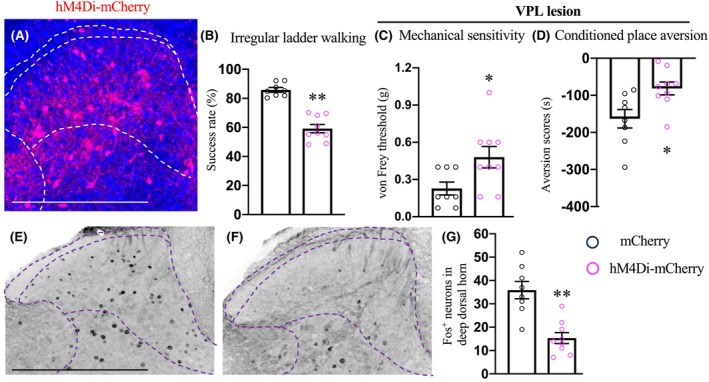
Effects of transient inactivation of the CST targeted lumbar spinal interneurons on the maintenance of CPSP. (A) A representative image of a transverse lumbar spinal dorsal horn stained with mCherry, demonstrating intersectional expression of hM4Di (*n* = 9, experimental) in CST targeted lumbar spinal interneurons. Intersectional expression of mCherry (*n* = 8) was used as control. Scale bar: 500 μm. (B) Performance of irregular ladder walking task in intact control and experimental mice. (C, D) Measurement of mechanical sensitivity at various time points (C) and aversion of tactile stimuli (D) in control and experimental mice receiving excitotoxic lateral thalamic lesions. (E–G) Transverse images of spinal sections stained for Fos (E, F) with quantifications of Fos^+^ neurons in the deep dorsal horn (G). Scale bar: 500 μm. **p < 0.01 or *p < 0.05, Student's *t*‐test.

Starting with chemogenetic silencing (Figure 5A), CNO administration led to decreased success rate in the irregular ladder walking task, consistent with the results from chronic inactivation of post‐synaptic targets of the CST (Figure 5B). Furthermore, chemogenetic inactivation of these neurons not only reduced mechanical hypersensitivity but also tactile evoked aversion in mice with CPSP (Figure 5C,D). Consistently, the activation of spinal neurons induced by touch was significantly reduced (Figure 5E–G). Thus, activation of spinal neurons receiving cortical input was required for the maintenance of the CPSP hypersensitivity.

### 3.6 Effects of chemogenetic silencing of post‐synaptic targets of the CST on evoking mechanical allodynia

**FIGURE 6:**
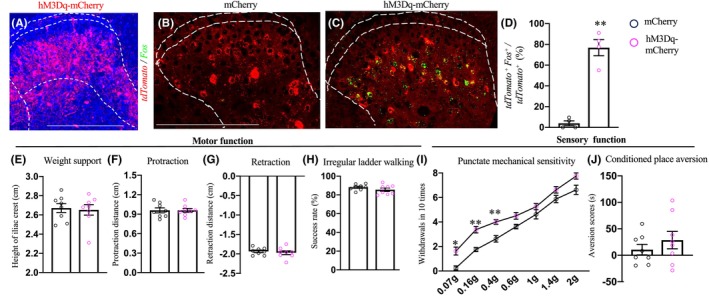
Effects of chemogenetic activation of the CST targeted lumbar spinal interneurons on mechanical sensitivity. (A) A representative image of a transverse lumbar spinal dorsal horn stained with mCherry, demonstrating intersectional expression of hM3Dq (*n* = 8, experimental) in CST targeted lumbar spinal interneurons. Intersectional expression of mCherry (*n* = 8) was used as control. (B–D) Representative images of fluorescent in situ hybridization of transverse spinal sections for *Fos* and *tdTomato* RNA expression (B,C) with quantification (D) from animals with post‐synaptic targets of CSNs labeled by mCherry or hM3Dq‐mCherry upon CNO injection, respectively. Scale bars: 500 μm. **p < 0.01, Student's *t*‐test. (E–H) Performance of ground walking (E–G), irregular ladder walking (H) task in intact control and experimental mice. (I, J) Measurement of mechanical sensitivity (I) and aversion of tactile stimuli (J) in intact control and experimental mice. **p < 0.01 or *p < 0.05, repeated measures of ANOVA followed by the Bonferroni correction.

Finally, we investigated whether chemogenetic activation of spinal interneurons targeted by hindlimb CSNs, confirmed by evoked Fos expression (Figure 6B–D), led to mechanical hypersensitivity (Figure 6A). CNO injection activated had no effects on gross locomotion, nor did it affect the performance on irregular ladder walking (Figure 6E–H).

We next measured mechanical sensitivity in mice with intersectional activation spinal neurons targeted by hindlimb CSNs. Upon CNO administration, we observed increased responses to innocuous (0.07, 0.16, and 0.4 g) but not nociceptive (above 0.6 g) von Frey filaments (Figure 6I). However, no mechanical allodynia, featured by hindpaw flinching, licking, and guarding behaviors,^37^, ^38^ has been evoked by chemogenetic activation of the total interneuron pool receiving direct cortical input. In light of this, innocuous tactile stimuli evoked no aversion, as demonstrated by the CPA test (Figure 6J). Thus, activation of post‐synaptic spinal targets of CSNs was insufficient to evoke mechanical allodynia, a prominent feature of CPSP seen in both human patients and rodent models,^5^, ^31^, ^39^ pointing to additional supraspinal origins of CPSP.

## 4 DISCUSSION

Although documented more than a century ago, there is a notable lack of both theoretical insight into the mechanisms and development of treatment options for CPSP. In current study, we first validated that a unilateral excitotoxic lesion of the VPL was sufficient to evoke prolonged mechanical pain hypersensitivity and activate corticospinal neurons (CSNs) and their spinal targets in the deep dorsal horn in response to innocuous tactile stimuli. Our results showed that activation of post‐synaptic spinal targets of CSNs was required for both the establishment and maintenance of mechanical hypersensitivity and pain perception of CPSP. The findings that a direct corticospinal pathway is involved in CPSP echoed the clinical observations and provided insights into the development of novel therapeutic interventions to treat this highly refractory central pain syndrome after stroke and other traumatic injuries.

Pain perception is continuously modulated by a fine balance between ascending spinothalamic tracts (STTs) and supraspinal descending circuits.^40^, ^41^ Disturbances of such equilibria have been proposed to be the underlying mechanisms of CPSP. Early in 1911, Head and Holmes proposed that injury to the lateral thalamus will lead to disinhibition of the medial thalamus.^42^ Such disinhibition theory has been further developed by following studies that define hyperactivity in the medial STT as neural substrates leading to CPSP. For instance, Craig et al. discovered that lesion in the lateral STT that transmits cool sensation leads to the disinhibition of a medial limbic network.^43^, ^44^ More recent studies showed that the disinhibition is not limited to the medial STT but could also occur in the thalamus, due to loss of normal ascending spinal inputs.^45^, ^46^ Our findings revealed that innocuous tactile stimuli following thalamic lesions enhanced neuronal activation in the somatosensory cortex, particularly in corticospinal neurons, thus revealing novel regions and cells involved in lesion‐induced disinhibition.

Accumulating clinical observations identified hyperactive bursting firing in the VPL in central pain patients.^47^, ^48^ Notably, when compared to patients with non‐CPSP chronic pain, micro‐stimulation of the VPL is more readily to evoke burning sensation.^49^, ^50^ Thus, lesion‐induced thalamic hyperexcitability is a prominent feature of human CPSP pathophysiology. Since somatosensory cortex including corticospinal neurons receives direct innervation from the VPL,^51^ we hypothesized that lesion activated somatosensory thalamus amplified the thalamocortical ascending pathway and ultimately resulted in overactivation of corticospinal neurons as observed in this study (Figure 2). In light of this, sparing of CST in CPSP patients correlates with cortical disinhibition and poor prognosis.^8^ Recent studies revealed that chronic neuropathic pain reduced activity of somatostatin‐expressing inhibitory neurons, leading to persistent elevation of layer V pyramidal neurons in the somatosensory cortex.^52^ Therefore, it will be intriguing to investigate how lateral thalamic lesions influence the activity of different types of cortical interneurons in the somatosensory cortex.

A previous study demonstrated that CPSP is transmitted through neither TRPV‐1 expressing sensory afferents nor NK1‐receptor expressing neurons in the superficial laminae.^31^ Consistently, Takami et al. showed that mechanical allodynia occurred following stroke is dependent on myelinated A fibers that project to deep dorsal horn, where CST axons terminate.^53^ Our observations that tactile stimuli evoked Fos activation in the deep but not superficial laminae supported this observation. Furthermore, silencing of post‐synaptic targets of CSNs reduced mechanical pain hypersensitivity, suggesting that overactivation of deep dorsal horn interneurons with direct cortical input was required for the development of CPSP, highlighting a crucial role of spino‐cortico‐spinal loop in CPSP pathophysiology.

CPSP, like other neuropathic disorders, are highly refractory, with only moderate treatment response by using a combination of multiple medications.^5^ Repetitive transcranial magnetic stimulation (rTMS) of the motor cortex is a non‐invasive treatment for refractory pain and has been utilized as a therapy for CPSP.^54^, ^55^ However, the precise mechanisms of its therapeutic effects remain unclear. Intriguingly, several studies reported that the effectiveness of rTMS in treating CPSP depends on the integrity of the CST axons.^56^, ^57^ Our findings highlighted the role of direct corticospinal modulation in CPSP pathophysiology, thereby shedding light on potential mechanisms that could explain the benefits of rTMS in treating this condition. In addition, our discoveries suggested that refined neurostimulation therapy protocols that are able to silence somatosensory corticospinal neurons could be powerful tools for treating CPSP and other central chronic pain conditions.

Our study has several limitations. First, although kainate injection effectively induces thalamic lesions, we have not explored the potential impacts of other types of thalamic lesions, such as a confined hemorrhagic lesion, on the activation of corticospinal neurons and their spinal targets. Second, we have not tested whether manipulation of spinal targets of CSNs had effects on cold allodynia, a prominent feature of CPSP. Future research aimed at identifying the molecular characteristics of CSN‐targeted spinal neurons will be crucial for elucidating the mechanisms by which the cortico‐spinal pathway mediates pain hypersensitivity following a stroke.

## AUTHOR CONTRIBUTIONS

S.H. conceived the experiments. F.F., T.Y., B.W., J.Z., J.D., and G.W. performed the experiments. F.F. and T.Y. prepared the manuscript with input from all authors.

## FUNDING INFORMATION

This work was supported by grants from National Natural Science Foundation (81501007) and the Tibet Natural Science Foundation (XZ2019ZR‐ZY41).

## CONFLICT OF INTEREST STATEMENT

The authors declare no competing interests.

## CONSENT TO PARTICIPATE

Not applicable.

## CONSENT FOR PUBLICATION

Not applicable.

## Data Availability

All original data will be made available on reasonable request to Shukun Hu.
